# Is catheter-tissue contact force value important for ablation of ventricular arrhythmias originating from the left ventricular papillary muscles?

**DOI:** 10.3389/fcvm.2023.1166810

**Published:** 2023-05-19

**Authors:** Jakub Baran, Martyna Skrzyńska-Kowalczyk, Roman Piotrowski, Agnieszka Sikorska, Tomasz Kryński, Piotr Kułakowski

**Affiliations:** ^1^ Division of Clinical Electrophysiology, Department of Cardiology, Centre of Postgraduate Medical Education, Grochowski Hospital, Warsaw, Poland; ^2^ Department of Internal Medicine and Cardiology University Clinical Center, Medical University of Warsaw, Warsaw, Poland

**Keywords:** antero-lateral papillary muscle, postero-medial papillary muscle, radiofrequency ablation, intracardiac echocardiography, contact force

## Abstract

**Background:**

Good catheter-tissue contact is mandatory to create effective ablation lesions. The minimal contact force value for ablation of arrhythmias originating from the left ventricle is 8.0–10.0 grams but is not known for arrhythmias arising from papillary muscles.

**Purpose:**

To analyze contact force values during successful ablation procedures of arrhythmias originating from the left ventricular papillary muscles.

**Methods:**

24 consecutive patients (mean age 57.9 ± 11.9 years, 16 males) underwent ablation of premature ventricular complexes originating from left ventricular papillary muscles with the use of CARTO electro-anatomical system and intracardiac echocardiography.

**Results:**

Acute complete abolition of ventricular ectopy was obtained in 23 (96%) patients. The fluoroscopy time was 3.9 ± 3.5 min and procedure duration - 114.8 ± 37.9 min. The mean contact force during successful ablations was 3.0 ± 1.1 grams and 3.18 ± 1.8 grams for antero-lateral and postero-medial papillary muscle, respectively (NS). The mean contact force during a single unsuccessful ablation was 3.0 grams. At control Holter ECG, the mean Ectopy Burden was Reduced in the Antero-Lateral Papillary Muscle Group from 18.0% ± 7.9% to 2.6% ± 2.9% (*p* = 0.005415) and in the Postero-Medial Papillary Muscle Group - from 34.8% ± 13.7%–1.7% ± 1.3% (*p *= 0.012694). During Median 27 (IQR: 17–34) Months of Follow-up There one Recurrence of Arrhythmia.

**Conclusion:**

The values of contact force for successful ablation of ventricular ectopy originating from the left ventricular papillary muscles may be much lower than those for ablation of other foci which questions the role of contact force measurement when ablating these arrhythmias.

## GRAPHICAL ABSTRACT

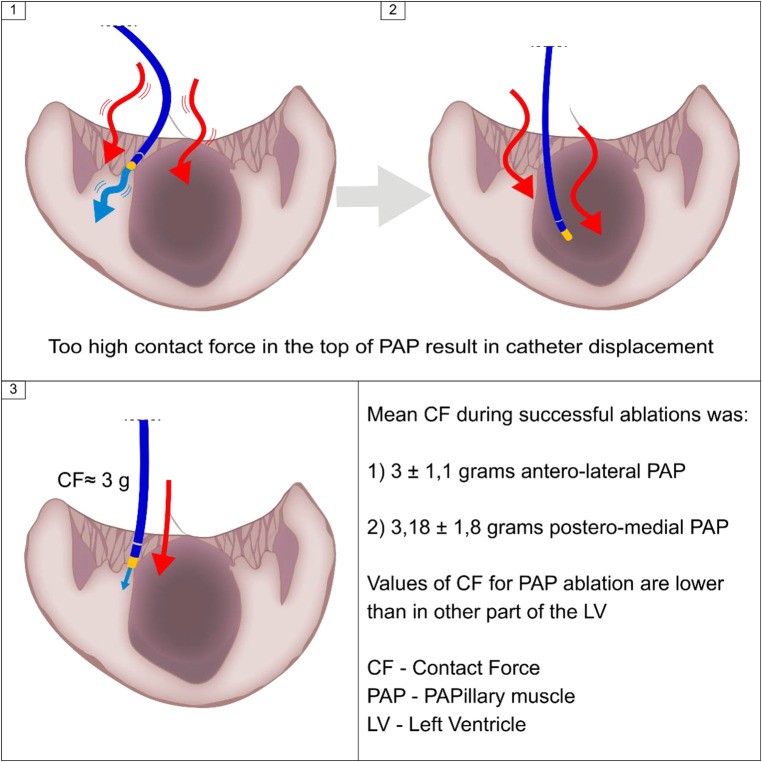

## Introduction

Efficacy of catheter radio-frequency (RF) ablation of premature ventricular contractions (PVC) depends on several factors, including power, duration, irrigation flow rate and contact force (CF) (1). Stable catheter–tissue contact with adequate CF is necessary for good energy coupling from the catheter to the tissue (2, 3). The importance of an appropriate real-time CF has been widely accepted (2–6). During mapping and ablation of arrhythmias originating from the left ventricle (LV) the minimal CF values of 8.0–10.0 g are recommended (7). However, for arrhythmias arising from the LV papillary muscles (PM), data on the role of sufficient CF value are limited.

Ablation of arrhythmias from LV PM is often difficult, because these structures are very mobile, stable catheter-tissue contact is uneasy to obtain, and ablation electrode often slides down from the head of PM when more firm contact is attempted. Thus, optimal or achievable CF values at these sites may be different from those in the other parts of LV.

Accordingly, the aim of this study was to analyse CF values during effective ablation of premature ventricular contractions (PVC) originating from LV PM.

## Methods

### Study population and data collection

The consecutive patients who underwent ablation for PVC arising from LV PM between May 2016 and June 2020 were included in this study. All procedural data were collected prospectively. Patients gave written informed consent to undergo ablation and to use their procedural, demographic and clinical data for research purposes. The study was approved by the local Ethical Committee of Centre of Postgraduate Medical Education (86/2022).

### Procedural protocol

All antiarrhythmic drugs were discontinued for at least 5 half-lives prior to the procedure. The procedures were performed under local anaesthesia. The choice of access (retrograde transaortic or transseptal) to the LV was left to the preference of the operator. For the transseptal approach, venous access was obtained via the right femoral vein. Following intracardiac echocardiography (ICE; ACUSON, AcuNav, Siemens Medical Solutions, Malvern, Pensylvania, USA) guided transseptal puncture, one 8.5 F SL1/SL0 sheath (Abbott, Saint Paul, Minnesota, USA) was advanced to the left atrium. For the retrograde approach, one SL0/SL1 sheath (Abbott, Saint Paul, Minnesota, USA) was inserted via the right femoral artery, and the end of the sheath was advanced just below left subclavian artery, which provided good catheter control and prevented the catheter “pop out” from the LV.

The procedures were performed under ICE guidance using the 3D CARTO system (Biosense Webster, Irvine, California, USA). Contact between the catheter and the myocardium was measured in grams (g). The calibration of the CF-sensing catheter was performed prior to mapping, with the catheter tip free in the LV, without contact with any cardiac structures, as assessed using ICE visualisation and lack of local intracardiac electrograms.

Based on the surface 12-lead ECG pattern either antero-lateral or postero-medial PM was firstly mapped as target location. If no perfect spot was found, additional area in LV was mapped. The left anterior oblique fluoroscopic view was used to orient the catheter tip, with position confirmed on ICE. Only patients with electrophysiological parameters confirming PM as a source of PVC (prematurity >15 ms in bipolar recording, no R wave in unipolar recording and other mapped sites with less encouraging parameters) were included in the study.

Ablation targets were the sites of the earliest activation and lack of R wave in the unipolar lead. The mean CF was stabilized over a few consecutive sinus beats before the onset of the radiofrequency application. The reason for it is high contractility of PM during RF-induced ventricular tachycardia, causing unpredictable catheter-to-tissue relation and unreliable CF values. Before each RF application the mean CF value was calculated. If RF application resulted in complete disappearance of clinical PVC from PM, the previously recorded CF was regarded as an effective value and used for the further analysis (Supplementary Movie #1). Before each application the electrode-myocardium contact was confirmed by ICE. The crucial role of ICE was demonstrated by Kalman et al. (8), who showed that an ablation catheter tip often slides along the heart surface during regular cardiac motion, despite fluoroscopic images suggesting fixed contact with cardiac wall and displaying local electrograms consistent with presumed good contact. Radiofrequency applications were delivered for a minimum of 30.0 s, with a mean power of 31.0 ± 1.5 W, and irrigation flow of 17.0 ml/min.

### Follow-up

Within 24 h of the procedure a resting 12-lead electrocardiogram was performed. All patients were seen in an out-patient clinic and at least one 24-hour Holter ECG was recorded. Patients' symptoms were also assessed. If reported symptoms were suspected to be associated with arrythmia recurrence, another 24-hour ECG monitoring was proposed. Effective ablation was defined as >95% reduction of PVC count compared with baseline pre-ablation Holter ECG, with a morphology identical to the ablated PVC.

### Statistical analysis

Continuous variables are presented as mean ± SD or median and interquartile ranges (IQR) as appropriate. Descriptive statistics for categorical data were expressed in absolute numbers and percentages. The Shapiro–Wilk test of normality was used to assess whether quantitative data conformed to the normal distribution. A 2-tailed *p*-value of <0.05 was considered to indicate statistical significance.

## Results

### Baseline characteristics

**Figure 1:**
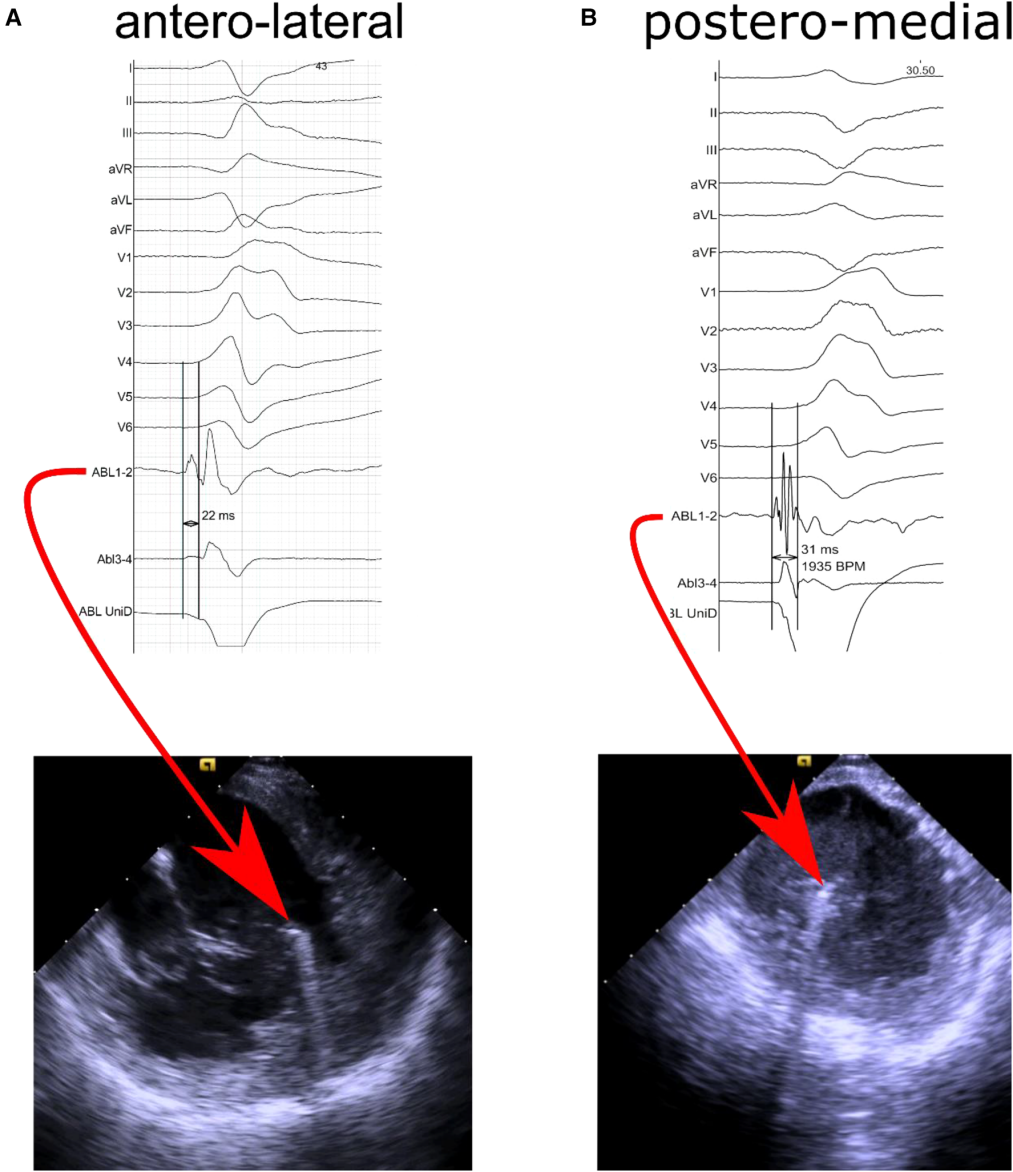
Representative intracardiac echocardiography images and intracardiac signals recorded from ablation electrode located (**A**) at the top of the antero-lateral papillary muscle with prematurity of 22 milliseconds in distal bipolar recording channel and no R wave in unipolar recording, and (**B**) at the top of the postero-medial papillary muscle with prematurity of 31 milliseconds in distal bipolar recording channel and no R wave in unipolar recording.

The baseline characteristics of the study population are depicted in Table 1. A total of 24 consecutive patients were included in this study (mean age 57.9 ± 11.9 years, 16 males); 5 patients underwent ablation for ischaemic PVC and 19 patients for idiopathic PVC. The mean ejection fraction was 50.63% ± 9.23%. There were 6 patients who underwent prior LV ablation. In 8 patients PVC originated from anterolateral PM, in 15 - from posteromedial PM, and 1 patient had PVC from both PM. In 23 (96%) subjects the source of PVC was located on the tip of PM (representative examples of prematurity of intracardiac signals recorded by ablation catheter located on tip of PM are presented in Figure 1; ablation for PVC originating from AL PM – Supplementary Movie #2; ablation for PVC originating from P-M PM – Supplementary Movie #3). Twelve patients had also PVC from other sites. Patients with PVC from posteromedial PM group had greater PVC burden than those with PVC coming from anterolateral PM.

**Table 1:** Baseline characteristics.

	All
Total	24
Male	16 (67%)
Age (years) (SD)	57.9 ± 11.9
BMI (SD)	29.2 ± 7.01
Previous ablation	6 (25%)
PVCs - frequency on 24 h Holter [%] (SD)	28.4 ± 14.5
- anterolateral papillary muscle (SD)	18.0 ± 7.9
- posteromedial papillary muscle (SD)	34.8 ± 13.7
- both papillary muscles (SD)	12.0 ± 0.0
PVCs – localization	
- anterolateral papillary muscle	8 (33%)
- posteromedial papillary muscle	15 (63%)
- both	1 (4%)
Other ectopies also present	12 (50%)
PVCs location on the tip of papillary muscle	23 (96%)
LVEF prior ablation [%] (SD)	50.63 ± 9.23
Ischemic PVCs	5 (21%)
Idiopathic PVCs	19 (79%)

### Procedural characteristics

All procedures were performed by the first author. Transaortic route was chosen 21 patients, while transseptal puncture was performed in 3 patients (Table 2). Fluoroscopy time was 3.9 ± 3.5 min and procedural duration - 114.8 ± 38 min. The mean of 8.74 ± 6.78 RF applications was performed with the mean application time of 518.5 ± 437.25 sec. Local bipolar electrograms showed 28.57 ± 7.64 msec of prematurity and His-Purkinje like signals were present at the ablation site in 38% of patients.

**Table 2:** Procedural characteristics.

	ALL
Retrograde approach	21 (88%)
Transseptal access	3 (12%)
ICE	24 (100%)
Procedural time [minutes] (SD)	114.8 ± 37.9
Application number (SD)	8.74 ± 6.78
Application time [seconds] (SD)	518.5 ± 437.25
Prematurity [miliseconds] (SD)	28.57 ± 7.64
Potentials of conduction system at the ablation site	9 (38%)
ICE loop at successful ablation site	23 (96%)
Mean CF during successful ablations [g] (SD)	3.1 ± 1.5
- anterolateral papillary muscle [g] (SD)	3.0 ± 1.1
- posteromedial papillary muscle [g] (SD)	3.18 ± 1.8
Mean CF during unsuccessful ablation [g] (SD)	3.0
- anterolateral papillary muscle [g] (SD)	3.0
- posteromedial papillary muscle [g] (SD)	0
Fluoroscopy [minutes] (SD)	3.9 ± 3.5
Fluoro dose [mGy/cm^2^] (SD)	1433,85 ± 1766,31

The mean CF during successful ablations was 3.1 ± 1.5 g, with 3.0 ± 1.1 g and 3.18 ± 1.8 g for anterolateral and posteromedial PM, respectively (*p* = 0.809975). The mean CF during an unsuccessful ablation was 3.0 g. The values of CF were similar in retrograde and transseptal approach (3.1 ± 1.6 g vs. 3.0 ± 1.1 g, *p* = 0.961089).

### Procedural outcome

Acute success was obtained in 23 (96%) patients (Table 3). The median follow-up duration was 27.4 months (IQR: 17.2–34.4). Out of 23 with acute procedural success, 1 (4%) patient experienced a recurrence. On the control Holter ECG the mean PVC burden was reduced in the whole study group from 28.4% ± 14.5% to 2.4% ± 2.6% (*p* = 0.000004); in the anterolateral PM group from 18.0% ± 7.9% to 2.6% ± 2.9% (*p* = 0.005415) and in the posteromedial PM group - from 34.8% ± 13.7% to 1.7% ± 1.3% (*p* = 0.012694).

**Table 3:** Acute success, effect on LVEF and recurrences.

	All
Acute success	23 (96%)
Median follow-up time [months]	27.4 (IQR 17.2–34.4)
PVCs - frequency on 24 h Holter [%]	2.4 ± 2.6
- anterolateral papillary muscle	2.6 ± 2.9
- posteromedial papillary muscle	1.7 ± 1.3
- both	3.6
Recurrence after acute successful procedure	1 (4%)
- anterolateral papillary muscle	1 (4%)
- posteromedial papillary muscle	0 (0%)

## Discussion

The main finding of our report is that CF values needed for successful RF ablation of PVC originating from LV PM slightly exceed 3.0 g and were much lower than recommended in other parts of LV. There are several possible reasons why the values of CF are lower when effectively ablating LV PM compared with other parts of LV.

Firstly, the most frequent site of origin of PVC is the head of PM (9), however, it is often difficult to stabilize ablation catheter in this area. If the contact force is too high the electrode slides off from the tip of PM toward its basal part, as kinetic friction force is always smaller than static friction force for a given pair of surfaces. The principal result of catheter lateral skating is that the same energy is delivered for a larger area, which compromises lesion efficacy (5, 10, 11). Secondary, catheter surface sliding may create unpredictable lesion dimensions, leading to ablation of structures adjacent to the target tissue (12–16). Another factor interfering with catheter stability is acceleration of ectopic activity during RF application - increase of the heart rate shortens diastole and reduces left ventricular diameter, which inevitably facilitates the catheter move from the target position toward the base of the PM. What can be done is to pull the catheter slightly back as previously demonstrated (9), especially in hearts with not reduced left ventricular ejection fraction like in our study.

Secondly, it seems that in these patients arrhythmia substrate lies superficially in endocardium, especially in those in whom His-Purkinje signals are recorded at the site of successful ablation. Thus, even if CF is rather low or catheter is sliding, effective lesions can be created. Although some of our patients had PVCs arising from other sites, it did not significantly alter the long-term success in contrast with available data (17). However, it was described that source of origin of PVC might be located deeply in the LV muscle, under the base part of the papillary muscle. In that situation higher contact force values are also an option for ablation (18).

Another explanation of successful ablation achieved with low CF is the possibility that CF values displayed by the system do not depict the real contact between PM and catheter. Very often ICE shows that catheter is in contact with cardiac tissue, yet CF values are low. This is particularly visible when the catheter is in parallel contact with PM.

Our data may also suggest that measuring CF during ablation of PVC originating from PM may be not as important as in other parts of LV. The same suggestion was presented by Lin et al. (17) who also used ICE and in their study the mean CF was 10.4 g for anterolateral PM and 11.6 g for posteromedial PM (19). Our data showed that even lower CF values were associated with effective ablation.

ICE imaging probably shows better true contact between PM and ablation catheter than mean CF values displayed by the system. As a consequence, it can be argued that catheters with CF measurement may not be required for PMs PVCs ablation. In refractory cases cryoenergy has been shown to be a valuable option due to more stable contact and lower arrhythmogenicity (19, 20).

Our strategy resulted in 8.74 ± 6.78 applications to achieve procedural endpoint. The mean RF time was comparable with what was previously reported (21–24).

We demonstrated that the overall median CF showed no significant differences between the retrograde and transseptal approach, in accordance with Tilz et al. (25) At the beginning we preferred the retrograde route, as in our opinion it offered better catheter stability (9), however, due to increasing number of patients with peripheral artery disease, both approaches became equally valuable. Since Qdot catheter (Biosense Webster) was presented for the everyday use, we speculate about its application for PVC ablation purpose. As presented by Heeger C et al. (26) it is reasonable to evaluate, whether this ablation catheter with the microeletrodes embanked on its tip will allow us to better understand of the direction of wavefront depolarization in the area of interest and more precisely depict a true source of arrhythmia.

There are several limitations of our study. Firstly, the study group was relatively low. Secondly, the use of steerable sheaths, which definitely affect the CF values, was not systematic. Thirdly, the study was observational and without a control group. However, we believe that our results may have impact on the choice of catheters when ablating PM in an everyday clinical practice.

## Conclusions

The values of contact force for successful ablation of ventricular ectopy originating from the left ventricular papillary muscles may be much lower than those for ablation of other foci which questions the role of contact force measurement when ablating these arrhythmias.

## Data Availability

The datasets presented in this article are not readily available. Requests to access the datasets should be directed to Jakub Baran jakub.baran1111@gmail.com.
